# Measurement Reliability for the Anatomical Characteristics of Cervical Muscles Using Musculoskeletal Ultrasound in Healthy Individuals

**DOI:** 10.3390/muscles4030028

**Published:** 2025-08-05

**Authors:** Georgios Sidiropoulos, Nikolaos Strimpakos, Asimakis K. Kanellopoulos, Maria Tsekoura, Konstantinos Alexiou, Olympia Papakonstantinou, Zacharias Dimitriadis

**Affiliations:** 1 Health Assessment and Quality of Life Research Laboratory, Department of Physiotherapy, Faculty of Health Sciences, University of Thessaly, 35132 Lamia, Greece; nikstrimp@uth.gr (N.S.); akanellopoulos@uth.gr (A.K.K.); zdimitriadis@uth.gr (Z.D.); 2 Therapeutic Exercise and Sports Rehabilitation Laboratory, Physiotherapy Department, University of Patras, 25100 Egio, Greece; mariatsekoura@upatras.gr; 3 Human Performance and Rehabilitation Laboratory, School of Health Sciences, University of Thessaly, 35132 Lamia, Greece; konstantalexiou@uth.gr; 4 2nd Department of Radiology, “Atticon” University General Hospital, National and Kapodistrian University of Athens, 11528 Athens, Greece; sogofianol@gmail.com

**Keywords:** ultrasound imaging, cervical spine, muscle morphology, reliability

## Abstract

Background: The reliable assessment of cervical muscle morphology is essential for both clinical and research use. However, evidence on the reliability of ultrasound measurements remains limited. Objective: To investigate the intra-rater and test–retest reliability of morphological measurements of the Longus Colli, Sternocleidomastoid, Multifidus Cervicis, and Semispinalis Capitis muscles using musculoskeletal ultrasound. Methods: Cross-sectional area, anteroposterior, and lateral dimensions were assessed using B-mode ultrasound. Anterior neck muscles were scanned in the supine position, while posterior neck muscles were scanned in the prone position. Each muscle was measured three times (to assess intra-rater reliability), which was repeated after 30 min (to assess test–retest reliability). Measurements were also normalized according to BMI and neck circumference. Results: Intra-rater reliability was found to be good to excellent for the Longus Colli (ICC = 0.77–0.92), excellent for the Sternocleidomastoid (ICC = 0.93–0.99), good to excellent for the Semispinalis Capitis (ICC = 0.89–0.97), and moderate to excellent for the Multifidus Cervicis (ICC = 0.69–0.92). Test–retest reliability was found to be moderate to good for the Longus Colli (ICC = 0.73–0.87), good to excellent for the Sternocleidomastoid (ICC = 0.84–0.98), good to excellent for the Semispinalis Capitis (ICC = 0.78–0.95), and good to excellent for the Multifidus Cervicis (ICC = 0.80–0.92). Conclusions: Musculoskeletal ultrasound demonstrates strong reliability for cervical muscle assessment, supporting its clinical use.

## 1. Introduction

Neck pain is the second most common musculoskeletal condition and the fourth leading cause of disability worldwide [1]. The annual prevalence in Europe is 6.5% [2]. Of these cases, 20% progress to chronic neck pain. Chronic neck pain is more frequently observed in women, with the most affected age group being 45–54 years [3]. Altered muscle function and morphological changes in these muscles are recognized features of painful neck disorders [4]. The assessment of muscles in the cervical region presents unique anatomical and technical challenges, including smaller muscle size, deeper location, and close proximity to vascular and neural structures, all of which may impact measurement precision. Understanding the morphology of deep cervical muscles such as the Longus Colli, Multifidus Cervicis, and Semispinalis Capitis is essential for evaluating movement dysfunction and pain in the cervical spine. These muscles belong to the local stability system, which is responsible for segmental control and maintaining neutral joint position [5,6,7].

Accurate and reliable examination of muscle morphology is essential for both clinical decision-making and research. Musculoskeletal ultrasound imaging has emerged as a widely used modality for assessing muscle quantity due to its non-invasive nature, accessibility, and cost-effectiveness. It allows for real-time visualization of muscle structure and is particularly useful for repeated assessments over time [8,9,10,11]. However, the utility of ultrasound is contingent on the consistency of its measurements, making the establishment of intra- and inter-rater reliability a critical prerequisite for its application. Recently, ultrasound automation and AI-based segmentation represent interconnected advances transforming medical imaging [12]. AI-driven solutions now facilitate automated image analysis, enabling precise lesion detection across various tissues. Javanshir et al. [13] reviewed 16 studies investigating the use of ultrasonography for assessing cervical muscles, which showed great potential despite identifying significant methodological considerations. They suggested using consistent landmarks, knowledge of anatomy and the function of target muscles, and the proper definition of muscular borders to help obtain a clearer image. Furthermore, they highlighted the use of standardized subject positioning, the correct placement of the transducer, and the use of multiple images for statistical analysis to improve results.

Normalization improves the validity of comparisons by controlling for body size, enabling clearer interpretation of muscle health or function. The normalization of ultrasonographic measurements with body mass index (BMI) is crucial, particularly when assessing neck muscles between healthy and pathological populations. However, there is a need to explore new methods of normalization, as current approaches may not adequately account for the unique characteristics of the cervical region.

Therefore, the aim of this study was to (a) determine the intra-rater and test–retest reliability of ultrasonographic measurements of anatomical characteristics of the Longus Colli, Sternocleidomastoid, Multifidus Cervicis, and Semispinalis Capitis in healthy individuals and (b) test the reliability of the same measurements after their normalization with BMI and neck circumference.

## 2. Materials and Methods

### 2.1. Sample

Sample size calculation was performed using an online calculator (https://wnarifin.github.io/ssc/ssicc.html, accessed on 8 February 2024) based on the guidelines by Walter et al. [14]. Sample size calculation showed that for a reliability study with two measurements after formulating an α level of 0.05 and a statistical power of 80%, the minimum required sample size is 28 participants (ρ_0_ = 0.5, ρ_1_ = 0.8). A convenience sample of 37 healthy individuals was finally recruited to achieve better accuracy in the estimation of reliability measures. Participants had to be between 18 and 35 years old and have a satisfactory cognitive level and language skills to provide their informed consent. Individuals with neck pain, other musculoskeletal disorders, or neurological impairments and/or a history of surgery in the cervical area were excluded from the study. The sampling was conducted via word of mouth among students of the University of Thessaly, as well as through responses from the local community of Lamia after advertising the study on social media. All participants gave written informed consent after they had time to read and understand the relevant information sheet. The study was conducted according to the guidelines of the Declaration of Helsinki and was approved by the Internal Deontology Committee of the Physiotherapy Department, University of Thessaly, Lamia, Greece (4524/24 March 2024).

### 2.2. Study Design

This was a reliability study, which was performed in two different sessions with a 30 min interval between them. The study was designed to assess the consistency of the recordings of the first session for calculating intra-rater reliability and the consistency of the recordings between the two sessions for calculating test–retest reliability. All measurements were performed at the “Health Assessment and Quality of Life Research Laboratory”.

### 2.3. Equipment and Materials

Ultrasound imaging was performed using the Versana Premier™ system (GE Healthcare, Milwaukee, WI, USA) with two linear probes. A linear probe (12L–RS) applied with a 38.4 mm footprint and a frequency of 8–15 MHz was used for the deep neck muscles. For the Sternocleidomastoid muscle, a high-performance linear array probe was used (GE L3-12-D Ultrasound Probe) with a 38 mm footprint, operating within a frequency range from 8 to 12.0 MHz. Ultrasound was used exclusively for recording the size of cervical muscles without providing any medical diagnosis or any other medical service. An inclinometer (Zebris, Isny im Allgäu, Germany) was used to ensure the successful performance of the first scanning process. Other materials used were a sterile skin marker (Medline Industries Ltd., Warrington, UK), blue ultrasound gel (Aquasonic, Parker Laboratories, Fiarfield, NJ, USA), powder-free gloves, ultrasound probe cover heads made from natural latex with lubricant (SoftCare, Bournas Medicals, Kifisia, Greece), and special cleaning wipes for ultrasound probes.

### 2.4. Procedure

The scanning was performed in B-mode. The radiological principle ALARA (as low as reasonably achievable) was used to obtain the muscle quantity characteristics, with a gain setting at a fixed 65 dB [15]. The frequency used for all the ultrasonographic scans of the Sternocleidomastoid muscle was high (12 MHz), whereas a lower frequency (8 MHz) was applied for the deeper muscles. The depth was set to 4.0 cm for all scans, whereas the focal depth was adjusted according to the specific location of each targeted muscle. Participants then lay on a plinth, and ultrasound imaging was performed in both supine and prone positions. For each target muscle, three images were acquired via transverse scan. Every scan was taken separately following the same procedure. In the first scanning process, the examiner marked the anatomical landmarks with a medical pen, using an inclinometer to ensure the parallel placement of the probe. After the completion of all the measurements in the four muscles, there was a 30 min resting period. After this period, during which participants remained in the lab and were asked not to engage in activities that could potentially influence the measurements, the procedure was repeated by the same examiner.

Ultrasonographic procedure for the anterior neck muscles

**Figure 1:**
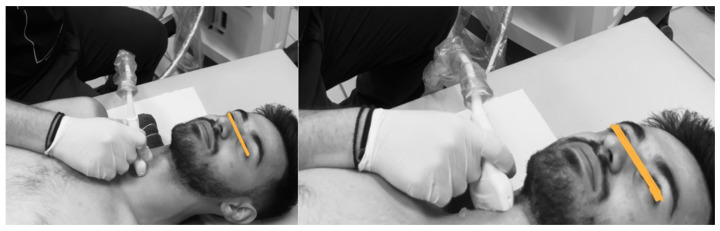
Ultrasonography of the anterior neck muscles (Longus Colli—**left**. Sternocleidomastoid—**right**).

All subjects were positioned supine in a standardized neutral posture, with cervical support provided by a small towel placed beneath the neck to maintain physiological lordosis (Figure 1). Upper extremities were positioned alongside the torso. Initial anatomical landmarks were established through manual palpation of the thyroid and cricoid cartilages, after which the ultrasound transducer was positioned transversely at the midline between these structures. Based on established anatomical references, the cricoid cartilage corresponds to the C6 vertebral level [16], whereas the bottom of the laryngeal prominence of the thyroid cartilage corresponds with the C5 level [17]. With the cricoid cartilage in the middle of the screen, the probe was moved approximately 2 cm laterally over the thyroid gland until the carotid artery was visible in the transverse view. The Longus Colli (LCo) was then visible between the carotid artery and the vertebral bodies (VBs). The thickness measurement of the LCo was taken from the midpoint of the ventral surface of the C6 vertebral body, defined as the posterior border of the muscle, to the ventral part of the muscle, at its border with the prefascial tissues, surrounding the carotid artery. At this level, the LCo is clearly distinguishable from adjacent muscles, such as the longus capitis, which runs more laterally and typically attaches to the anterior tubercle of C6, thus reducing the risk of measurement error caused by muscle overlap [8]. To scan the Sternocleidomastoid muscle, the initial procedure used for the Longus Colli was repeated. The transducer was then advanced cranially to locate the C3–C4 spinal segment. Upon reaching this level, lateral probe adjustment was performed until the transducer’s central axis corresponded with the ipsilateral tragus alignment.

Ultrasonographic procedure for the posterior neck muscles

**Figure 2:**
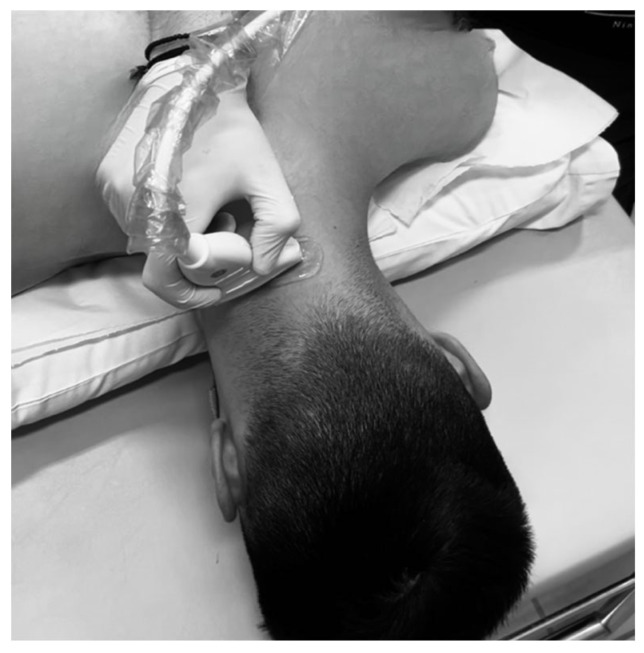
Ultrasonographic measurements of the posterior neck muscles.

After the completion of the assessment of the two anterior neck muscles, the participants were positioned in the prone position. Foreheads were placed onto the adjustable headpiece of the plinth with slight head flexion (Figure 2). The Multifidus Cervicis was measured at the C4 level. The examiner initially located the C7 vertebra through palpation [18] and then proceeded superiorly to identify the C4 level. The C4 vertebral level is considered optimal for ultrasonographic assessment of the Multifidus Cervicis muscle due to its anatomical accessibility. At this level, the Multifidus muscle is clearly distinguishable from adjacent muscles, as it is positioned superior to the rotators, medial to the Semispinalis muscles, and inferior to the splenius muscle [19]. Subsequently, the Semispinalis Capitis was assessed at the C3 level. The C3 vertebral level is considered optimal for ultrasonographic evaluation of the Semispinalis Capitis muscle due to its consistent anatomical appearance and high measurement reliability [20]. At C3, the muscle presents a clearer separation from adjacent cervical extensors such as Splenius Capitis and Semispinalis Cervicis, allowing for more precise delineation of its borders [21].

### 2.5. Data Processing

**Figure 3:**
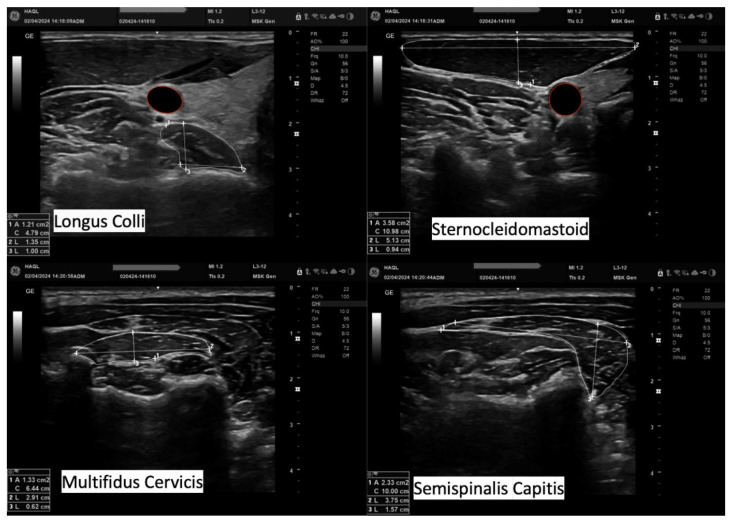
Data processing of the ultrasonographic measurements. Cross-sectional area, anteroposterior dimension, and lateral dimension of the Longus Colli, Sternocleidomastoid, Multifidus Cervicis, and Semispinalis Capitis.

After completing the data collection phase, all ultrasound images were stored and processed by the same examiner approximately one month later. At that time, the datasets were anonymized and coded to reduce observer bias during data processing. The CSA of each muscle was captured by tracing the perimeter of the muscle tissue carefully, excluding the surrounding fascial layers and connective tissues. The LD measurements were taken at their greatest visible distance, with the lines aligned, whereas APD was measured in the same way so that a 90-degree angle was formed between them (Figure 3).

### 2.6. Data Analysis

For descriptive statistics, means were used as measures of central tendency and standard deviations as measures of dispersion. Intra-rater and test–retest reliability was assessed based on the Intraclass Correlation Coefficient (ICC_2,1_), Standard Error of Measurement (SEM), and Smallest Detectable Difference (SDD). ICC values of 0–0.5, 0.5–0.75, 0.75–0.9, and 0.9–1 indicated poor, moderate, good, and excellent reliability, respectively [22]. SEM was calculated by extracting the square root of the residual mean square and was additionally expressed as a percentage of the grand mean. The calculation of the SDD was achieved by utilizing the formula SEM*1.96* square root of 2 and was additionally expressed as a percentage (%) of the grand mean. SDD values under 30% were regarded as adequate, whereas those under 10% were classified as exceptional; SEM values below 10% were considered adequate, whereas those below 5% were classified as exceptional [23]. IBM SPSS for MacOS, version 29.0 (IBM Corp., Armonk, NY, USA), was used for all statistical analyses.

## 3. Results

### 3.1. Demographics

In total, 37 participants (25 males and 12 females) completed this study. All data analyzed were normally distributed (Kolmogorov–Smirnov test: *p* > 0.05). The descriptive statistics and the quantitative ultrasound measurements of the participants’ demographics are presented in Table 1.

### 3.2. Intra-Rater Reliability of Anatomical Characteristics of Cervical Muscles

The study showed moderate to excellent intra-rater reliability for the recording of the anatomical characteristics of the cervical muscles with musculoskeletal ultrasound (ICC = 0.69–0.99). Standard Error of Measurement (SEM) % was found to range between 1.76–13.66%, and Smallest Detectable Difference (SDD) % was found to range between 4.84–37.64%. The findings of the study about the intra-rater reliability of musculoskeletal ultrasound for recording the muscle size of cervical muscles are analytically presented in Table 2.

### 3.3. Intra-Rater Reliability of Anatomical Characteristics of the Cervical Muscles Standardized Using Body Mass Index

The study showed good to excellent Intra-rater reliability for the recording of anatomical characteristics of the cervical muscles with musculoskeletal ultrasound, when these results were standardized using body mass index (BMI) (ICC = 0.77–0.99). SEM% was found to range between 1.84 and 15.32%, and SDD% was found to range between 4.26 and 41.57%. These findings are more analytically presented in Table 3.

### 3.4. Intra-Rater Reliability of Anatomical Characteristics of the Cervical Muscles Standardized Using Neck Circumference

The results also showed moderate to excellent intra-rater reliability for the anatomical characteristics of the cervical muscles when their size was standardized using neck circumference (NC) (ICC = 0.65–0.98). SEM% was found to range from 2.02–13.66% and SDD% was found to range from 5.11–37.69%. More details are presented in Table 4

### 3.5. Test–Retest Reliability of Anatomical Characteristics of the Cervical Muscles

The results of this study have shown good to excellence test–retest reliability for the anatomical characteristics of the cervical muscles with musculoskeletal ultrasound (ΙCC = 0.75–0.98), with the percentage of the Standard Error of Measurement (SEM%) ranging from 3.69–18.67%, and percentage of the Smallest Detectable Difference (SDD%) ranging from 1.78–51.13%. More details are presented in Table 5

### 3.6. Test–Retest Reliability of Anatomical Characteristics of the Cervical Muscles Standardized Using Body Mass Index

The results of this study have shown moderate to excellent test–retest reliability for the anatomical characteristics of the cervical muscles with musculoskeletal ultrasound standardized using body mass index (BMI) (ΙCC = 0.74–0.97), with the percentage of the Standard Error of Measurement (SEM%) ranging from 3.57–17.07%, and percentage of the Smallest Detectable Difference (SDD%) ranging from 9.85–46.75%. More details are presented in Table 6.

### 3.7. Test–Retest Reliability of Anatomical Characteristics of the Cervical Muscles Standardized Using Neck Circumference

The results of this study have shown moderate to excellence test–retest reliability for the anatomical characteristics of the cervical muscles with musculoskeletal ultrasound standardized using neck circumference (NC) (ΙCC = 0.57–0.95), with the percentage of the Standard Error of Measurement (SEM%) ranging from 3.87–18.51%, and percentage of the Smallest Detectable Difference (SDD%) ranging from 10.74–51.33%. More details are presented in Table 7.

## 4. Discussion

The aim of this study was to examine the reliability of musculoskeletal ultrasound in measuring the size of the Longus Colli, Sternocleidomastoid, Semispinalis Capitis, and Multifidus Cervicis. The intra-rater reliability was good for measuring the CSA of the Longus Colli and excellent for the CSA of the Sternocleidomastoid, Multifidus Cervicis, and Semispinalis Capitis. The APD showed moderate reliability for the Multifidus Cervicis, good reliability for the Longus Colli, and excellent reliability for the Sternocleidomastoid and Semispinalis Capitis. The LD was good for the Longus Colli, Multifidus Cervicis, and Semispinalis Capitis, whereas it showed excellent reliability for the Sternocleidomastoid. On the other hand, the test–retest reliability showed excellent reliability for the Semispinalis Capitis and Sternocleidomastoid, but good reliability for measuring the CSA of the Longus Colli and Multifidus Cervicis. The APD was found to be good for the Longus Colli, Multifidus Cervicis, and Semispinalis Capitis, and it was excellent for measuring the APD of the Sternocleidomastoid muscle. LD measurements were good for all muscles.

The observed variations in test–retest reliability may be attributed to inconsistent probe placement, as the retest process did not include the use of an inclinometer. Normalization shows minimal effects on intra-rater reliability for most of the morphological characteristics tested. However, normalization using BMI tends to provide better overall consistency for measurements of the CSA among the deep neck muscles, whereas neck circumference normalization had a more pronounced effect on the examination of dimensions, especially on the Multifidus Cervicis muscle. To our knowledge, this was the first time that an ultrasonographic study used neck circumference for normalization, and it seems that it can be successfully applied to the assessment of the size of neck muscles, as it appears to be more relevant to this specific anatomical region.

Previous research has examined the reliability of ultrasound measurements for the Longus Colli (LCo) muscle, with varying results across studies. Cross-sectional area (CSA) measurements have shown reliability ranging from moderate to excellent. One study of 27 healthy participants [17] reported moderate intra-rater reliability (ICC = 0.71, 95% CI: 0.57–0.81) when measuring at the C5–C6 level. However, other studies have found stronger reliability. Javanshir et al. [24] demonstrated good reliability (ICC = 0.90–0.93) in 15 subjects, while their subsequent study [25] of 20 subjects showed excellent reliability (ICC = 0.95). Additional research has supported these positive findings, with one study [26] reporting good reliability (ICC = 0.90, 95% CI: 0.94–0.98), and Nagai et al. [27] finding good reliability (ICC = 0.84, 95% CI: 0.63–0.94) in 24 participants. All reviewed studies measured the LCo muscle at the C5–C6 cervical level with participants in the supine position, matching our study’s methodology. None of the reviewed studies normalized their ultrasonographic findings; therefore, direct comparisons to our data could not be made.

Although previous studies have examined the APD of muscles in a supine position, they used a different procedure and performed the scanning at a different cervical level. In a study by Pirri et al. [9], the APD of the Sternocleidomastoid was found by using the landmarks of the carotid artery and internal jugular vein in the middle of the neck, under the mandibular bone. In total, 16 healthy participants were assessed, and the results for intra-rater reliability showed good reliability (ICC = 0.89, 0.85–0.92). In another study on healthy participants [28], the intra-rater reliability of measuring the APD of the Sternocleidomastoid was good (ICC = 0.88) from a resting position. The procedure used to find the cervical level for scanning ran halfway along the distance from the mastoid bone to the clavicular margin, referring to the C5-C6 level. In another study [29] on 30 healthy participants, the intra-rater reliability of measuring the APD of the Sternocleidomastoid was moderate for the right (ICC = 0.65) and good for the left side (ICC = 0.85) at the C5 level.

For the measurement of the Semispinalis Capitis, two studies have examined its intra-rater reliability. The first study [30] found excellent reliability (ICC = 0.93) when the scanning was performed at C2 and examining the APD of SSCap at rest, while the second study [8] found good reliability between the first three scans (ICC = 0.89, 0.75–95). For the intra-rater reliability of morphological characteristics of the Multifidus Cervicis, one study [8] used the C5 level and found good reliability for measuring CSA (ICC = 0.81, 0.70–0.89) in healthy participants, whereas another study [31] used the C4 level and found excellent reliability (ICC = 0.97, 0.92–0.98) in healthy participants. Both studies used the prone position. On the other hand, one study by Rahnama et al. [32] used a sitting position for measuring the thickness of the Multifidus Cervicis and found good reliability (ICC = 0.89) in healthy participants. The lack of agreement between some of these studies and our data was due to a loss of clarity of the fascial layer between the Semispinalis Cervicis and Multifidus muscles; most of the studies also recognize this challenge.

In the intra-rater reliability analysis for cervical muscle characteristics, the SEM values exhibited variability across different muscles. In the Longus Colli, the SEM for CSA was relatively low at 0.1 cm^2^, indicating a stable measurement, whereas the lateral dimension showed higher variability, with an SEM of 0.15 cm. The Sternocleidomastoid displayed relatively consistent values, with SEMs for CSA and lateral dimension at 0.1 cm^2^ and 0.08 cm, respectively. The Semispinalis Capitis and Multifidus Cervicis also demonstrated moderate variability in the lateral dimensions, with SEMs ranging from 0.16 cm in the Semispinalis Capitis to 0.11 cm in the Multifidus Cervicis. When muscle characteristics were normalized by BMI, the SEM values generally decreased, reflecting improved reliability. For example, the Longus Colli CSA showed a significant reduction in SEM, with a value of 0.003 cm^2^ compared to the non-normalized SEM of 0.1 cm^2^ (9.61%). This trend was consistent across other muscle characteristics. On the other hand, when the muscle characteristics were normalized using neck circumference, the SEM values generally increased compared to BMI normalization. For instance, the SEM for the Longus Colli CSA increased to 0.005 cm^2^ (18.51%) from 0.1 cm^2^ (9.61%). Similar patterns were observed for the Multifidus Cervicis and Semispinalis Capitis. This suggests that while neck circumference standardization provides some adjustments for muscle size variability, it introduces greater variability.

When applying our predefined thresholds (SEM < 10% and SDD < 30% for adequate reliability; SEM < 5% and SDD < 15% for exceptional reliability), distinct patterns emerged across muscles and measurement conditions. Exceptional reliability (SEM < 5%, SDD < 15%) was achieved exclusively by the Sternocleidomastoid muscle measurements, specifically for the anteroposterior dimension (SEM: 1.78%, SDD: 4.85%), lateral dimension (SEM: 1.76%, SDD: 4.84%), CSA (SEM: 2.77%, SDD: 7.69%), and APD/LD ratio (SEM: 3.04%, SDD: 8.38%). When standardized using BMI, these measurements maintained exceptional performance. Adequate reliability (SEM < 10%, SDD < 30%) was met by multiple measurements. All Sternocleidomastoid measurements, Semispinalis Capitis (CSA: SEM 6.24%, SDD 16.76%; APD: SEM 6.04%, SDD 16.58%; LD: SEM 4.41%, SDD 11.70%), and most of the Longus Colli measurements, except the APD/LD ratio, exceeded adequate thresholds. The Multifidus Cervicis showed mixed results, with the lateral dimension achieving adequate reliability (SEM: 4.39%; SDD: 11.33%) while the anteroposterior dimension failed both thresholds (SEM: 10.72%; SDD: 28.42%). These results show that most ultrasonographic measurements had low measurement errors and can be applied in clinical practice as a reliable method for examining neck muscle size.

### 4.1. Limitations

One limitation of this study is the use of a non-blind examiner, which may introduce bias in the data processing of the measurements. The same examiner who performed the data collection also conducted the data processing using the US system measurement tools, such as the region of interest and a caliper. To limit this bias, all data processing work took place at least one month after the data collection phase and was stored under code names. Another limitation of the present study was that test–retest reliability was performed on the same day, 30 min after the initial measurements. Although test–retest reliability over one or more days is generally preferable due to the reduction in possible testing effects such as memory of previous responses, practice effects, and fatigue, we decided to perform the retest session on the same day for several practical and methodological reasons. First, this approach minimized the possibility of potential history effects, including changes in participants’ physical or psychological states or exposure to external events that could influence their responses. Second, same-day testing reduced the risk of participant drop-out, which could have introduced attrition bias and reduced our sample size. While we acknowledge that this same-day approach may have inflated our reliability estimates due to recall bias and insufficient time for true stability assessment, we believe this decision represented the best balance between methodological rigor and practical feasibility given our study constraints. Furthermore, the ultrasound procedure was very passive and therefore no significant learning or fatigue effects are expected. Another limitation was the lack of an inter-rater reliability examination. The authors acknowledge that inter-rater reliability is crucial when multiple raters make subjective judgments. High inter-rater reliability would demonstrate that different raters reach similar conclusions, thus strengthening the validity of the results. Another limitation of this study is that the sample was restricted to healthy young adults, which may limit the generalizability of the findings to clinical populations, such as those with chronic musculoskeletal pain. However, the examination of the reliability of musculoskeletal ultrasound in measuring the muscle size of cervical muscles in healthy people was the necessary first step, as the resulting error in the measurement can be attributed exclusively to the procedure and the raters and is free from the error induced by the clinical condition of musculoskeletal pain in patients.

### 4.2. Clinical Implications

The findings from this study highlight the value of musculoskeletal ultrasound in assessing the morphological characteristics of cervical muscles, providing a reliable tool for time-efficient and easy clinical applications. Its high intra-rater and test–retest reliability, particularly for recording CSA and dimensions, suggest that ultrasound can be used effectively to monitor muscle size changes in clinical settings, such as in rehabilitation programs for neck pain or dysfunction. Additionally, the inclusion of normalization techniques, particularly using neck circumference, provides the opportunity to assess the relative size of cervical muscles, which is very useful when comparing the muscle size of cervical muscles between individuals or populations with different anthropometric characteristics. To improve reliability, the authors recommend consistent use of anatomical landmarks, a thorough understanding of the structure and function of the target muscles, and clear delineation of muscle borders to enhance image clarity. They also emphasize the importance of standardized subject positioning, correct transducer placement, and acquiring multiple images to strengthen the robustness of statistical analyses.

### 4.3. Recommendations for Future Research

Our study provides clinicians with a recommended ultrasound scanning protocol for assessing three deep neck muscles—the Longus Colli, Semispinalis Capitis, and Multifidus Cervicis—as well as a practical method for examining the local CSA of the Sternocleidomastoid. This approach may be helpful in future studies aiming to compare deep versus superficial anterior neck muscles. On the other hand, tools such as the inclinometer, which facilitate optimal probe placement, and practical training on a digital scale for applying consistent force during probe positioning, were two new approaches that proved helpful during the data collection phase. In terms of reliability, both intra-rater reliability and test–retest reliability were comparable. Based on this, we recommend that for future studies, the same examiner perform all ultrasound examinations, particularly if repeated scans are required for each individual muscle. Additionally, we recommend using more anatomical landmarks to identify cervical levels. Future research should further verify the reliability of ultrasound for measuring the morphological characteristics (CSA, APD, LD, and ratios) of cervical muscles, especially in clinical populations such as patients with chronic conditions. Building upon current studies, it is crucial to extend the exploration of reliability to other key cervical muscles to further understand the generalizability and robustness of musculoskeletal ultrasound in this context. Additionally, studies that incorporate normalized muscle size measurements, particularly in randomized controlled trials, will offer valuable insights. These approaches may reveal different patterns or trends that could inform clinical practice. Investigating these aspects in depth could lead to more nuanced conclusions regarding the role of ultrasound in assessing cervical muscle health, with implications for improving diagnostic accuracy and treatment protocols.

## 5. Conclusions

The results of this study suggest that musculoskeletal ultrasound is a reliable imaging tool to assess the morphological characteristics of various important neck muscles in a healthy adult population. The clinical importance of musculoskeletal ultrasound is reflected in its high reproducibility. The methodology for measurements and normalizations as implemented in our study might be further applied in studies with patients with neck pathologies. Findings from such studies could enhance the assessment capabilities at our disposal in order to optimize the clinical management of these patients.

## Figures and Tables

**Table 1:** Demographic characteristics and quantitative US measurements of the sample (n = 37; males/females = 25/12).

Characteristics	M	SD
Age (y)	21.76	±2.71
Height (cm)	175.8	±7.3
Weight (kg)	77.5	±13.5
Body Mass Index (BMI)	24.9	±3.6
Neck Circumference (cm)	37.07	±3.24
Longus Colli—CSA—(cm^2^)	1.05	±0.26
Longus Colli—APD (cm)	0.91	±0.15
Longus Colli—LD (cm)	1.64	±0.30
Sternocleidomastoid—CSA—(cm^2^)	3.68	±0.80
Sternocleidomastoid—APD (cm)	0.98	±0.18
Sternocleidomastoid—LD (cm)	4.78	±0.32
Semispinalis Capitis—CSA—(cm^2^)	2.49	±0.59
Semispinalis Capitis—APD (cm)	1.18	±0.30
Semispinalis Capitis—LD (cm)	3.79	±0.47
Multifidus Cervicis—CSA—(cm^2^)	1.41	±0.31
Multifidus Cervicis—APD (cm)	0.78	±0.13
Multifidus Cervicis—LD (cm)	2.69	±0.34

CSA: Cross-sectional area, APD: Anteroposterior dimension, LD: Lateral dimension, cm^2^: Centimeters squared, and cm: Centimeters.

**Table 2:** Intra-rater reliability of musculoskeletal ultrasound for recording anatomical characteristics of the cervical muscles.

Muscles	Characteristics	GM	ICC	95% CI	SEM%	SDD%
Longus Colli	CSA (cm^2^)	1.04	0.85	0.77–0.92	9.61%	26.65%
	Anteroposterior dimension (cm)	0.89	0.80	0.69–0.88	7.94%	21.80%
	Lateral Dimension (cm)	1.67	0.77	0.65–0.86	9.46%	24.89%
	APD/LD ratio	0.567	0.87	0.78–0.93	13.66%	37.64%
Sternocleidomastoid	CSA (cm^2^)	3.601	0.98	0.97–0.99	2.77%	7.69%
	Anteroposterior dimension (cm)	0.97	0.99	0.98–0.99	1.78%	4.85%
	Lateral Dimension (cm)	4.75	0.93	0.88–0.96	1.76%	4.84%
	APD/LD ratio	0.205	0.99	0.98–0.99	3.04%	8.38%
Semispinalis Capitis	CSA (cm^2^)	2.48	0.93	0.89–0.96	6.24%	16.76%
	Anteroposterior dimension (cm)	1.17	0.94	0.91–0.97	6.04%	16.58%
	Lateral Dimension (cm)	3.79	0.89	0.81–0.93	4.41%	11.70%
	APD/LD ratio	0.308	0.97	0.94–0.98	6.57%	18.17%
Multifidus Cervicis	CSA (cm^2^)	1.41	0.90	0.84–0.94	7.09%	19.65%
	Anteroposterior dimension (cm)	0.78	0.69	0.54–0.81	10.72%	28.42%
	Lateral Dimension (cm)	2.69	0.88	0.81–0.93	4.39%	11.33%
	APD/LD ratio	0.294	0.89	0.81–0.94	10.75%	29.22%

CSA: Cross-sectional area, APD: Anteroposterior dimension, LD: Lateral dimension, cm^2^: Centimeters squared, cm: Centimeters, APD/LD: Anteroposterior/Lateral dimension; GM: Grand mean, ICC: Intraclass Correlation Coefficient, SEM: Standard Error of Measurement, SDD: Smallest Detectable Difference.

**Table 3:** Intra-rater reliability of musculoskeletal ultrasound for recording anatomical characteristics of the cervical muscles, standardized using body mass index (BMI).

Muscles Standardized Using BMI	Characteristics	GM	ICC	95% CI	SEM%	SDD%
Longus Colli	CSA (cm^2^)	0.04	0.85	0.75–0.91	7.12%	20.78%
	Anteroposterior dimension (cm)	0.03	0.77	0.64–0.86	9.66%	26.79%
	Lateral Dimension (cm)	0.06	0.81	0.70–0.89	10%	27.71%
	APD/LD ratio	0.02	0.90	0.82–0.94	15.32%	41.57%
Sternocleidomastoid	CSA (cm^2^)	0.14	0.98	0.97–0.99	2.76%	5.93%
	Anteroposterior dimension (cm)	0.03	0.99	0.98–0.99	5.64%	12.62%
	Lateral Dimension (cm)	0.19	0.98	0.97–0.99	1.84%	4.26%
	APD/LD ratio	0.0083	0.99	0.97–0.99	3.29%	9.01%
Semispinalis Capitis	CSA (cm^2^)	0.09	0.90	0.84–0.94	6.57%	15.39%
	Anteroposterior dimension (cm)	0.04	0.94	0.89–0.96	7.11%	13.85%
	Lateral Dimension (cm)	0.15	0.87	0.79–0.93	4.05%	11.08%
	APD/LD ratio	0.012	0.97	0.96–0.99	6.61%	16.8%
Multifidus Cervicis	CSA (cm^2^)	0.05	0.88	0.80–0.93	8%	22%
	Anteroposterior dimension (cm)	0.03	0.77	0.67–0.87	11.54%	27.71%
	Lateral Dimension (cm)	0.109	0.92	0.87–0.96	4.49%	10.17%
	APD/LD ratio	0.012	0.93	0.89–0.96	12.07%	32.33%

CSA: Cross-sectional area, APD: Anteroposterior dimension, LD: Lateral dimension, cm^2^: Centimeters squared, cm: Centimeters, APD/LD: Anteroposterior/Lateral dimension; GM: Grand mean, ICC: Intraclass Correlation Coefficient, SEM: Standard Error of Measurement, SDD: Smallest Detectable Difference.

**Table 4:** Intra-rater reliability of anatomical characteristics of the cervical muscles standardized using neck circumference (NC).

Muscles Standardized Using NC	Characteristics	GM	ICC	95% CI	SEM%	SDD%
Longus Colli	CSA (cm^2^)	0.028	0.85	0.76–0.91	7.14%	27.71%
	Anteroposterior dimension (cm)	0.024	0.74	0.61–0.85	8.33%	23.09%
	Lateral Dimension (cm)	0.044	0.80	0.66–0.87	9.64%	26.45%
	APD/LD ratio	0.015	0.88	0.80–0.94	13.66%	37.69%
Sternocleidomastoid	CSA (mm^2^)	0.098	0.97	0.94–0.98	2.92%	7.91%
	Anteroposterior dimension (cm)	0.026	0.98	0.97–0.99	1.88%	5.11%
	Lateral Dimension (cm)	0.12	0.94	0.90–0.97	2.02%	5.54%
	APD/LD ratio	0.0056	0.98	0.97–0.99	3.14%	8.41%
Semispinalis Capitis	CSA (mm^2^)	0.066	0.89	0.81–0.94	5.86%	15.90%
	Anteroposterior dimension (cm)	0.031	0.92	0.87–0.96	6.03%	16.09%
	Lateral Dimension (cm)	0.102	0.77	0.65–0.87	4.15%	11.41%
	APD/LD ratio	0.0083	0.96	0.93–0.98	6.50%	18.03%
Multifidus Cervicis	CSA (mm^2^)	0.038	0.86	0.78–0.92	7.20%	19.69%
	Anteroposterior dimension (cm)	0.021	0.65	0.49–0.79	11.36%	30.35%
	Lateral Dimension (cm)	0.072	0.88	0.81–0.93	4.56%	12.31%
	APD/LD ratio	0.0080	0.90	0.83–0.94	12.5%	33.75%

CSA: Cross-sectional area, APD: Anteroposterior dimension, LD: Lateral dimension, cm^2^: Centimeters squared, cm: Centimeters, APD/LD: Anteroposterior/Lateral dimension; GM: Grand mean, ICC: Intraclass Correlation Coefficient, SEM: Standard Error of Measurement, SDD: Smallest Detectable Difference.

**Table 5:** Test–retest reliability of anatomical characteristics of the cervical muscles.

Muscles	Characteristics	GM	ICC	95% CI	SEM%	SDD%
Longus Colli	CSA (cm^2^)	1.03	0.75	0.52–0.87	18.67%	51.13%
	Anteroposterior dimension (cm)	0.906	0.82	0.65–0.91	10.47%	28.75%
	Lateral Dimension (cm)	1.63	0.87	0.74–0.93	10.26%	27.21%
	APD/LD ratio	0.57	0.83	0.67–0.91	12.40%	34.52%
Sternocleidomastoid	CSA (cm^2^)	3.66	0.98	0.96–0.99	4.73%	12.87%
	Anteroposterior dimension (cm)	0.977	0.93	0.86–0.96	6.47%	1.78%
	Lateral Dimension (cm)	4.77	0.84	0.69–0.92	3.69%	9.87%
	APD/LD ratio	0.204	0.88	0.77–0.94	8.05%	21.56%
Semispinalis Capitis	CSA (cm^2^)	2.503	0.95	0.91–0.98	6.91%	18.8%
	Anteroposterior dimension (cm)	1.15	0.89	0.77–0.94	11.33%	31.30%
	Lateral Dimension (cm)	3.78	0.87	0.75–0.93	6.14%	16.86%
	APD/LD ratio	0.305	0.78	0.58–0.89	14.66%	39.98%
Multifidus Cervicis	CSA (cm^2^)	1.43	0.85	0.71–0.92	12.70%	34.89%
	Anteroposterior dimension (cm)	0.79	0.82	0.65–0.91	9.80%	27.01%
	Lateral Dimension (cm)	2.69	0.88	0.77–0.94	5.99%	16.48%
	APD/LD ratio	0.29	0.85	0.86–0.93	10.90%	29.63%

CSA: Cross-sectional area, APD: Anteroposterior dimension, LD: Lateral dimension, cm^2^: Centimeters squared, cm: Centimeters, APD/LD: Anteroposterior/Lateral dimension; GM: Grand mean, ICC: Intraclass Correlation Coefficient, SEM: Standard Error of Measurement, SDD: Smallest Detectable Difference.

**Table 6:** Test–retest reliability of anatomical characteristics of the cervical muscles standardized using body mass index (BMI).

Muscles Standardized Using BMI	Characteristics	GM	ICC	95% CI	SEM%	SDD%
Longus Colli	CSA (cm^2^)	0.041	0.74	0.50–0.87	17.07%	46.75%
	Anteroposterior dimension (cm)	0.036	0.81	0.63–0.90	9.62%	25.82%
	Lateral Dimension (cm)	0.066	0.89	0.80–0.94	10.49%	28.97%
	APD/LD ratio	0.023	0.86	0.73–0.93	12.45%	33.74%
Sternocleidomastoid	CSA (cm^2^)	0.147	0.97	0.94–0.98	5.10%	13.38%
	Anteroposterior dimension (cm)	0.039	0.91	0.82–0.95	6.78%	18.47%
	Lateral Dimension (cm)	0.194	0.97	0.95–0.99	3.57%	9.85%
	APD/LD ratio	0.0083	0.85	0.71–0.92	7.80%	20.03%
Semispinalis Capitis	CSA (cm^2^)	0.100	0.92	0.85–0.96	7%	19%
	Anteroposterior dimension (cm)	0.046	0.86	0.73–0.93	11.29%	30.43%
	Lateral Dimension (cm)	0.152	0.85	0.71–0.92	6.17%	16.95%
	APD/LD ratio	0.012	0.82	0.65–0.91	13.17%	34.64%
Multifidus Cervicis	CSA (cm^2^)	0.058	0.83	0.67–0.91	12.19%	32.75%
	Anteroposterior dimension (cm)	0.032	0.89	0.89–0.94	9.05%	24.25%
	Lateral Dimension (cm)	0.109	0.93	0.87–0.97	6.15%	17.03%
	APD/LD ratio	0.012	0.92	0.84–0.96	10.54%	27.71%

CSA: Cross-sectional area, APD: Anteroposterior dimension, LD: Lateral dimension, cm^2^: Centimeters squared, cm: Centimeters, APD/LD: Anteroposterior/Lateral dimension; GM: Grand mean, ICC: Intraclass Correlation Coefficient, SEM: Standard Error of Measurement, SDD: Smallest Detectable Difference.

**Table 7:** Test–retest reliability of anatomical characteristics of the cervical muscles standardized using neck circumference (NC).

Muscles Standardized Using NC	Characteristics	GM	ICC	95% CI	SEM%	SDD%
Longus Colli	CSA (mm^2^)	0.027	0.73	0.47–0.86	18.51%	51.33%
	Anteroposterior dimension (cm)	0.024	0.76	0.54–0.88	10.03%	27.71%
	Lateral Dimension (cm)	0.044	0.87	0.74–0.93	10.41%	27.27%
	APD/LD ratio	0.015	0.84	0.70–0.92	12.82%	35.11%
Sternocleidomastoid	CSA (mm^2^)	0.097	0.95	0.91–0.98	4.94%	13.43%
	Anteroposterior dimension (cm)	0.026	0.86	0.74–0.93	6.66%	18.12%
	Lateral Dimension (cm)	0.129	0.87	0.74–0.93	3.87%	10.74%
	APD/LD ratio	0.0055	0.83	0.66–0.91	8.13%	22.17%
Semispinalis Capitis	CSA (mm^2^)	0.066	0.90	0.81–0.95	7.10%	19.31%
	Anteroposterior dimension (cm)	0.031	0.79	0.59–0.89	11.63%	32.18%
	Lateral Dimension (cm)	0.102	0.57	0.16–0.78	6.27%	17.39%
	APD/LD ratio	0.0082	0.69	0.40–0.84	13.35%	37.18%
Multifidus Cervicis	CSA (mm^2^)	0.038	0.80	0.61–0.90	12.10%	33.55%
	Anteroposterior dimension (cm)	0.021	0.81	0.64–0.90	9.40%	25.07%
	Lateral Dimension (cm)	0.073	0.89	0.78–0.94	5.97%	16.32%
	APD/LD ratio	0.0081	0.88	0.77–0.94	10.54%	29.08%

CSA: Cross-sectional area, APD: Anteroposterior dimension, LD: Lateral dimension, cm: Centimeters, APD/LD: Anteroposterior/Lateral dimension; GM: Grand mean, ICC: Intraclass Correlation Coefficient, SEM: Standard Error of Measurement, SDD: Smallest Detectable Difference.

## Data Availability

Data are unavailable due to privacy and ethical restrictions.
